# Retrospective analysis of bevacizumab‐induced hypertension and clinical outcome in patients with colorectal cancer and lung cancer

**DOI:** 10.1002/cam4.701

**Published:** 2016-04-25

**Authors:** Aya Nakaya, Takayasu Kurata, Takashi Yokoi, Shigeyoshi Iwamoto, Yoshitaro Torii, Yuichi Katashiba, Makoto Ogata, Madoka Hamada, Masanori Kon, Shosaku Nomura

**Affiliations:** ^1^ First Department of Internal MedicineKansai Medical UniversityOsakaJapan; ^2^ Department of SurgeryKansai Medical UniversityOsakaJapan

**Keywords:** Avastin^®^, bevacizumab, colorectal cancer, hypertension, non‐small cell lung cancer

## Abstract

Bevacizumab(Avastin^®^), a humanized therapeutic monoclonal antibody that targets vascular endothelial growth factor, is widely used in cancer treatment. Patients who are treated with bevacizumab have an increased risk of developing systemic hypertension. However, the relationship between bevacizumab‐induced hypertension and clinical outcome remains unclear. We aimed to evaluate the effect of bevacizumab‐induced hypertension in terms of prognosis in patients with colorectal cancer and non‐small cell lung cancer. The study included 632 patients, 317 patients with non‐small cell lung cancer and 315 patients with colorectal cancer. All patients were treated with bevacizumab in combination with standard chemotherapy protocols, between April 2007 and December 2014. Blood pressure was measured before each treatment cycle. In the patient group with colorectal cancer, treated with bevacizumab, Grade 2–3 hypertension was present in 27.6%. In hypertensive patients with colorectal cancer, median overall survival was 42.6 months, compared with 20.6 months for normotensive patients in this group (*P *= 0.00071). In the patient group with non‐small cell lung cancer, treated with bevacizumab, Grade 2–3 hypertension was present in 20.5%. In hypertensive patients with non‐small cell lung cancer, median overall survival was 43.0 months, compared with 26.3 months for normotensive patients in this group (*P *= 0.00451). Patients who developed hypertension during treatment with bevacizumab for colorectal cancer and non‐small cell lung cancer had significantly prolonged overall survival when compared with normotensive patients. Bevacizumab‐induced hypertension may represent a biomarker for clinical benefit in cancer patients treated with bevacizumab.

## Introduction

Bevacizumab is a humanized therapeutic monoclonal antibody that targets vascular endothelial growth factor (VEGF) and is widely used in current cancer treatments. In 2004, bevacizumab was the first angiogenesis inhibitor approved by the US Food and Drug Administration (FDA) as a first‐line treatment for metastatic colorectal cancer (CRC). In 2006, the FDA approved bevacizumab, in combination with carboplatin and paclitaxel, for the initial treatment of patients with unresectable, locally advanced, metastatic or recurrent, nonsquamous, non‐small cell lung cancer (NSCLC).

Bevacizumab treatment is associated with several recognized adverse events including gastrointestinal perforations, delayed wound healing, hemorrhage, arterial thrombosis, proteinuria, reversible posterior leukoencephalopathy syndrome, neutropenia and infection, and congestive heart failure. Bevacizumab is also associated with the onset of hypertension (HTN), with 11%–16% of patients requiring treatment for HTN while being treated with bevacizumab 1, 2. Bevacizumab‐induced HTN is one of the most documented adverse events associated with bevacizumab treatment and is usually managed by standard oral hypertensive medications and rarely results in discontinuation of oncology treatment.

Studies of the VEGF pathway have revealed the mechanism by which bevacizumab affects blood pressure. It is thought to involve decreased production of nitric oxide (NO). VEGF increases NO synthesis, and VEGF inhibition diminishes NO synthesis. Because NO is a vasodilator, decreased NO leads to constriction of the vasculature and a reduction in sodium ion renal excretion, which ultimately leads to increased blood pressure 3, 4, 5, 6.

Hypothesizing that the onset of bevacizumab‐induced HTN during treatment involves successful inhibition of the VEGF pathway, studies have examined the relationship between HTN and outcome among patients treated with bevacizumab. In the correlative study accompanying the Eastern Cooperative Oncology Group (ECOG) 4599 study of NSCLC, patients with bevacizumab‐induced HTN experienced significantly longer overall survival (OS) than patients without bevacizumab‐induced HTN 7. A significant improvement in treatment response and time to progression has also been reported for patients with metastatic CRC who required antihypertensive medication during treatment with bevacizumab 8, 9, 10. In contrast, a meta‐analysis of six studies and five controlled clinical trials that included 5900 patients with a variety of cancers, found that bevacizumab‐induced HTN during treatment was not predictive of clinical benefit or prognosis; however, in one trial, newly developed bevacizumab‐induced HTN was predictive of improved progression‐free survival and overall survival (OS) in the bevacizumab group compared with a control group 11. Therefore, the relationship between bevacizumab‐induced HTN and clinical outcome remains unclear.

We aimed to determine whether the frequency and severity of bevacizumab‐induced HTN were different for different tumor types and whether bevacizumab‐induced HTN is associated with a better prognosis in patients treated with bevacizumab both in CRC and NSCLC.

## Patients and Methods

A retrospective study was performed of patients who were treated with bevacizumab for CRC between April 2007 and December 2014, and of patients who were treated with bevacizumab for NSCLC between 2009 and 2014, at the Kansai Medical University Hirakata Hospital, Japan. Eligible patients had advanced, metastatic, or recurrent nonsquamous NSCLC or CRC. Exclusion criteria included a history of significant hemoptysis, radiological evidence of tumor invading or abutting major blood vessels, and a history of thrombotic or hemorrhagic disorders. Evidence of central nervous system (CNS) metastases was an initial exclusion criterion, but several clinical reports revealed that cerebral hemorrhage was not necessarily associated with CNS metastases 12, 13. Therefore, from 2012, patients with asymptomatic CNS metastases were enrolled. This retrospective clinical study was conducted in accordance with the Declaration of Helsinki, and local institutional review board requirements.

### Treatment

Patients with NSCLC received bevacizumab at a dose of 15 mg/kg every 3 weeks in association with combination chemotherapy. Patients with CRC received bevacizumab at a dose of 5 mg/kg as a first‐line therapy or 10 mg/kg as second‐line therapy every 2 weeks, in association with cytotoxic chemotherapy.

### Study assessments

Patient treatment response was evaluated according to Response Evaluation Criteria In Solid Tumors 14 by whole‐body computerized tomography performed every 8–12 weeks. Blood pressure was measured before each treatment cycle. In all patients with elevated baseline blood pressure, this was medically controlled during the chemotherapy.

The evaluation of HTN was based on the Common Toxicity Criteria for Adverse Events version 4.0 (CTCAE v4.0) 15, but adapted for this study as follows: Grade 1: transient, asymptomatic, blood pressure increase, and no therapy required; Grade 2: recurrent, persistent, or symptomatic increase requiring monotherapy at any time during bevacizumab therapy; Grade 3: requiring more than one drug therapy or more intensive therapy than Grade 2. For patients with a history of HTN and who were on a stable regimen of antihypertensive therapy, if their blood pressure rose above baseline requiring a change to a more powerful antihypertensive therapy, they were defined as Grade 2.

### Statistical analysis

An important aim of this study was to determine whether there was any correlation between bevacizumab‐induced HTN and OS. Patient OS was calculated from the start of bevacizumab‐containing chemotherapy until the time of death or the last clinical follow‐up. Survival curves were created using the Kaplan–Meier method, and differences were evaluated using the log‐rank test. A stepwise multivariate logistic regression analysis was performed to evaluate the factors associated with bevacizumab‐induced HTN. All statistical tests were two‐sided, and statistical significance was set at *P* < 0.05, and 95% confidence intervals were calculated. All statistical analyses were performed using EZR (Saitama Medical Center, Jichi Medical University, Saitama, Japan), which is a graphical user interface for R version 2.13.0 (The R Foundation, Vienna, Austria). More precisely, it is a modified version of R Commander (version 1.6–3) designed to add the statistical functions frequently used in biostatistics.

## Results

### Patients

The clinical characteristics of the patients in this study are shown in Table 1. A total of 632 patients were identified, including 317 patients with NSCLC and 315 patients with CRC. There was no difference in the sex and median age between CRC and NSCLC patients.

**Table 1:** The patient's characteristics

Pt characteristics	Colon		Lung	
*n*	315		317	
Sex(%)				
Male	54		66	
Female	46		34	
Median Age(y/o)(range)	66 (36–88)		68 (34–86)	
Stage(%)				
I	1		10	
II	10		5	
III	34		22	
IV	55		63	
Median survival time(months)(range)	26.3(21.5–31.3)		30.0(24.0–38.5)	
Median Treatment durations(months)(range)	9.4 (1–58.6)		8.8(1–54.6)	
Median Bevacizumab cycles(*n*)(range)	12 (1–83)		7 (1–36)	
Combined regimes(%)	mFOLFOX6	35	carboplatin‐based	56
	XELOX	26	pemetrexed	31
	DeGramont	23	taxane	5
	FOLFIRI	16	others	8

CRC, colorectal cancer; NSCLC, non‐small cell lung cancer; modified FOLFOX, oxaliplatin and fluorouracil/leucovorin; XELOX, oxaliplatin and capecitabine; DeGramont, fluorouracil/leucovorin; FOLFIRI, irinotecan and fluorouracil/leucovorin.

The patients with CRC received a median of 12 cycles of bevacizumab and the patients with NSCLC received a median of six cycles of bevacizumab. The median treatment time for patients with CRC was 9.3 months and the median treatment time for patients with NSCLC was 8.8 months. The median survival time for patients with CRC was 26.3 months and the median survival time for patients with NSCLC was 30.0 months. For patients with NSCLC, the most common combined regimen was carboplatin‐based (56%), followed by pemetrexed (31%), a taxane (5%), and others (8%). Most NSCLC patients were treated with bevacizumab as maintenance therapy. In patients with CRC, the most common combined regimen was modified FOLFOX‐6 (oxaliplatin and fluorouracil/leucovorin; 35%), followed by XELOX (oxaliplatin and capecitabine; 26%), de Gramont (fluorouracil/leucovorin; 23%), and FOLFIRI (irinotecan and fluorouracil/leucovorin; 16%). In CRC, most patients were treated with bevacizumab beyond disease progression and until an event occurred resulting in cessation of treatment.

### Frequency of adverse events

In the patients studied, the most common bevacizumab‐related adverse event (AE; ≤Grade 2) was bevacizumab‐induced HTN (23.8%). HTN was diagnosed in 27.6% of the patients with CRC and 20.5% of the patients with NSCLC. In 20.4% of patients in the study, there was a history of HTN, and these patients were on antihypertensive medication before chemotherapy began.

**Figure 1:**
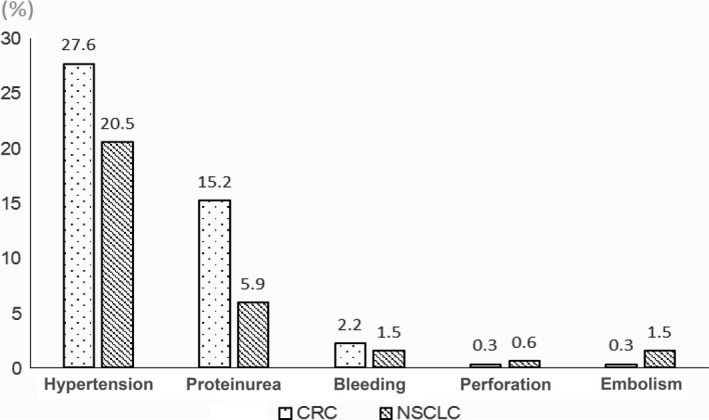
Frequency of adverse events followed by proteinuria and bleeding. These major adverse events occurred more frequently in patients with colorectal cancer than in patients with non‐small cell lung cancer.

The other adverse events (AEs) in the bevacizumab‐treated patients were proteinuria (10.3%), and bleeding (1.8%). These AEs were more common in patients with CRC than NSCLC (Fig. 1). The most common serious adverse events (≤Grade 3) were bleeding (2.0%), thromboembolism (1.1%), and perforation (0.4%; Table 2). Gastrointestinal perforation was rare (0.4%), but most of these cases were fatal (mortality >60%). Bleeding was associated with organ involvement by cancer. Bleeding in patients with NSCLC more commonly involved the upper airway. Bleeding in patients with CRC more commonly involved the gastrointestinal tract. Thromboembolism was more common in patients with NSCLC and was associated with deep‐vein thrombosis (DVT). The incidence of fatal AEs was 1.26% in NSCLC and 0.63% in CRC.

**Table 2:** The frequency of serious adverse events

	Tumor type	Age	Sex	Regimen	Bev cycles	Grade
Perforation	NSCLC	63	M	CBDCA+PEM	4	G5
		69	M	CBDCA+PEM	2	G3
	CRC	48	F	FOLFOX	5	G5
Bleeding	NSCLC	74	F	CBDCA+PEM	7	G3(hemoptysis)
		68	M	CBDCA+PEM	3	G5(hemoptysis)
		63	M	CBDCA+PEM	3	G3(GI)
		75	M	CBDCA+PEM	1	G5(hemoptysis)
		73	M	CBDCA+PEM	1	G3(GI)
	CRC	80	M	FOLFOX	5	G3(nasal)
		68	M	XELOX	30	G3(GI)
		67	M	FOLFOX	12	G3(GI)
		60	M	FOLFOX	3	G3(nasal)
		71	M	XELOX	83	G3(GI)
		64	M	FOLFOX	6	G3(GI)
		60	M	FOLFIRI	16	G3(GI)
Embolism	NSCLC	76	F	CBDCA+PEM	5	G3
		59	M	CBDCA+PEM	12	G5
		42	F	CBDCA+PEM	6	G3
		77	F	CBDCA+PEM	8	G3
		65	M	CBDCA+PEM	7	G3
	CRC	71	F	FOLFIRI	7	G5
		72	M	DeGramont	39	G3

CRC, colorectal cancer; NSCLC, non‐small cell lung cancer; CBDCA, carboplatin; PEM, pemetexed; FOLFOX, oxaliplatin and fluorouracil/leucovorin; XELOX, oxaliplatin and capecitabine; DeGramont, fluorouracil/leucovorin; FOLFIRI, irinotecan and fluorouracil/leucovorin.

### Hypertension and overall survival

**Figure 2:**
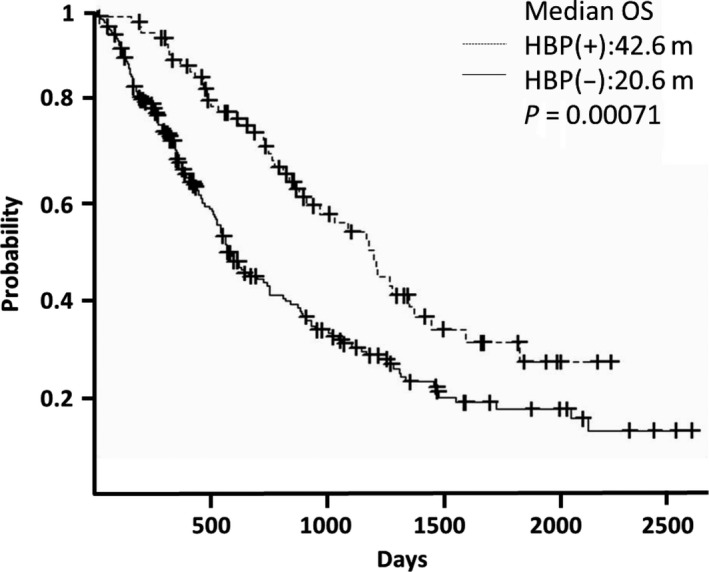
Hypertension and overall survival in colorectal cancer.In colorectal cancer, median overall survival of patients with hypertension was 42.6 months, whereas in normotensive patients it was 20.6 months (*P* = 0.00071).

**Figure 3:**
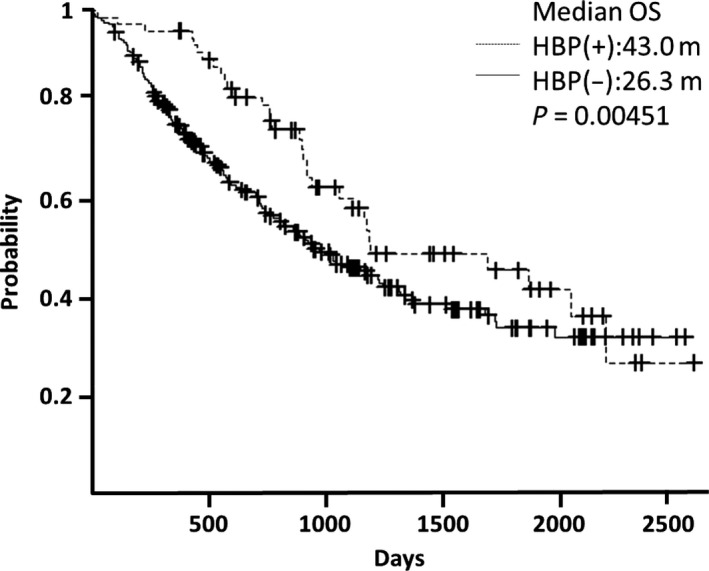
Hypertension and overall survival in non‐small cell lung cancer. In non‐small cell lung cell cancer, median overall survival of patients with hypertension was 43.0 months, whereas, in normotensive patients it was 26.3 months (P = 0.00451).

Patients with bevacizumab‐induced HTN had a significantly prolonged OS in both CRC and NSCLC. In CRC, the median OS of patients with bevacizumab‐induced HTN was 42.6 months; for normotensive patients, it was 20.6 months (*P* = 0.00071; Fig. 2). In NSCLC, the median OS for patients with bevacizumab‐induced HTN was 43.0 months; for normotensive patients, it was 26.3 months (*P* = 0.00451; Fig. 3).

The onset of bevacizumab‐induced HTN occurred after three cycles of bevacizumab in NSCLC, and after six cycles in CRC. These results indicate that bevacizumab‐induced HTN developed it in the early cycles of bevacizumab treatment, as median cumulative bevacizumab treatment cycles were seven in NSCLC patients and 12 in CRC patients.

### Multivariate analysis

In this study, we attempted to investigate other factors that may be associated with the incidence of HTN, such as age over 65 years, sex, cumulative bevacizumab cycles, past history of HTN, therapy duration of greater than 6 months, and the clinical complication of proteinuria.

The multivariate analysis showed that the more than 10 cumulative cycles of bevacizumab treatment was associated with a significantly increased risk for HTN in patients with NSCLC. In contrast, bevacizumab therapy duration for more than 6 months was associated with a significantly increased risk for HTN in patients with CRC. In patients with both types of cancer, a history of HTN was not associated with an increased risk of developing HTN during bevacizumab treatment (Table 3).

**Table 3:** Multivariate analysis

	CRC			NSCLC		
	Odds ratio	95% CI	*P*	Odds ratio	95% CI	*P*
Age						
≥65 years	1.2	0.703–2.050	0.502	1.01	0.57–1.77	0.98
<65 years	1			1		
Sex						
Male	1.18	0.69–2.01	0.552	0.682	0.384–1.210	0.191
Female	1			1		
Bevacizumab cycles						
≥10 cycles	1.71	0.716–4.08	0.227	1.06	1.02–1.10	0.003
<10 cycles	1			1		
Past history of hypertension						
Hypertension(+)	1.82	0.97–3.380	0.059	1.4	0.73–2.69	0.31
Hypertension(−)	1			1		
Therapy duration						
≥6 months	2.89	1.12–7.41	0.0275	1.1	0.511–2.38	0.802
<6 months	1			1		
Complication with proteinurea						
Proteinurea+	1.02	0.587–1.770	0.964	1.2	0.592–2.440	0.611
Proteinurea−	1			1		

CRC, colorectal cancer; NSCLC, non‐small cell lung cancer.

## Discussion

This retrospective clinical study aimed to evaluate the effect of bevacizumab‐induced HTN in terms of prognosis in patients with CRC and NSCLC. In this study, both CRC and NSCLC patients who developed HTN during bevacizumab treatment had significantly prolonged OS when compared with normotensive bevacizumab‐treated CRC and NSCLC patients. Furthermore, it was noted that bevacizumab‐treated patients who did develop HTN did so during early treatment cycles. The results of this study indicate that early development of bevacizumab‐induced HTN may be a predictive marker for the efficacy of bevacizumab treatment in these cancer patients.

There have been some previous reports that support our findings and which have shown that early development of HTN was predictive of outcome among patients with NSCLC and CRC. Dahlberg et al. showed that bevacizumab‐induced HTN occurring within 1 month of bevacizumab treatment for lung cancer is predictive for patient survival 7. Österlund et al. showed HTN developing within 3 months of bevacizumab treatment had an independent prognostic effect in patients with CRC 9. Mir et al. reported that in patients with NSCLC, CRC, or ovarian cancer, early development of HTN within 42 days of bevacizumab treatment was predictive of treatment response 16. These published results contradict other published studies that have reported that prolonged exposure to bevacizumab increased the risk of developing HTN 17, 18.

We hypothesize that there were two response groups; in one group, patients receiving long‐term bevacizumab treatment who may develop late‐onset HTN, and in the other group are patients who develop early‐onset HTN. In both groups, the development of bevacizumab‐induced HTN may prolong survival. If prolonged exposure to bevacizumab increases the risk of developing HTN, then either cumulative cycles of treatment or therapy duration reflect bevacizumab dose intensity, and may correlate with bevacizumab‐induced HTN. In the multivariate analysis done in this study, cumulative cycles of bevacizumab treatment (over 10 cycles) were correlated with HTN only in patients with NSCLC, whereas bevacizumab treatment duration over more than 6 months was correlated with HTN in patients with CRC.

It is possible that bevacizumab‐induced HTN and early‐onset bevacizumab‐induced HTN patient groups may have overlapped in our multivariate analysis. Furthermore, median treatment cycles were longer for CRC patients, therefore, using a study cutoff at 10 treatment cycles may not be adequate to reveal statistically significant differences. This same explanation might apply to using a study cutoff at 6 months for NSCLC patients because median treatment duration was shorter than for CRC patients. In this study, we initially assumed that proteinuria and a previous history of HTN might be risk factors for developing bevacizumab‐induced HTN, but the multivariate analysis did not support this. We also assumed that proteinuria, which is one of most common AEs of bevacizumab, might affect OS. However, it was associated only with NSCLC, not with CRC(data not shown).

This study had some limitations. It was performed retrospectively and involved a single treatment center in Japan. The endpoint of the study was OS, and not progression‐free survival, which may imply some ambiguity in the evaluation of the patient responses. Furthermore, there is no consensus by the CTCAE as to which blood pressure measurement should be recorded. We performed blood pressure measurements only at the beginning of each treatment cycle. Blood pressure was measured in a hospital clinic environment rather than a home environment. All these considerations may have resulted in overdiagnosis of HTN in the patients included in this study.

In conclusion, the most common adverse event associated with bevacizumab treatment for patients with CRC and NSCLC is bevacizumab‐induced HTN. The findings of this study have shown that in both these patient groups, patients who developed HTN while on bevacizumab treatment had significantly improved OS compared with normotensive bevacizumab‐treated patients. This result indicates a connection between bevacizumab‐induced HTN and patient outcome in both CRC and NSCLC. We recommend larger randomized studies to evaluate the role of bevacizumab‐induced HTN as a potential prognostic biomarker in cancer patients treated with bevacizumab.

## Conflict of Interest

None reported.
